# The Effect of Larval Diet on Adult Survival, Swarming Activity and Copulation Success in Male *Aedes aegypti* (Diptera: Culicidae)

**DOI:** 10.1093/jme/tjx187

**Published:** 2017-10-04

**Authors:** Bethan J Lang, Stefano Idugboe, Kirelle McManus, Florence Drury, Alima Qureshi, Lauren J Cator

**Affiliations:** 1 Grand Challenges in Ecosystems and Environment, Department of Life Sciences, Imperial College London, United Kingdom; 2 Department of Animal and Environmental Biology, University of Benin, Benin City, Nigeria

**Keywords:** *Aedes aegypti*, immature diet, ecology & behavior, mosquito control, mating strategy

## Abstract

Control of *Aedes aegypti* (L.) (Diptera: Culicidae) populations is vital for reducing the transmission of several pervasive human diseases. The success of new vector control technologies will be influenced by the fitness of laboratory-reared transgenic males. However, there has been relatively little published data on how rearing practices influence male fitness in *Aedes* mosquitoes. In the laboratory, the effect of larval food availability on adult male fitness was tested, using a range of different fitness measures. Larval food availability was demonstrated to be positively correlated with adult body size. Larger males survived longer and exhibited greater swarming activity. As a consequence, larger males may have more mating opportunities in the wild. However, we also found that within a swarm larger males did not have an increased likelihood of copulating with a female. The outcome of the mating competition experiments depended on the methodology used to mark the males. These results show that fitness assessment can vary depending on the measure analyzed, and the methodology used to determine it. Continued investigation into these fitness measures and methodologies, and critically, their utility for predicting male performance in the field, will increase the efficiency of vector control programs.

The mosquito *Aedes aegypti* (L.) (Diptera: Culicidae) is a vector of several pervasive human diseases, such as Dengue fever and Zika virus, for which there are currently no widely deployed vaccines (Tolle 2009, Petersen et al. 2016). While insecticides have been used successfully to control *Aedes* populations in the past, the evolution of insecticide resistance threatens many of the conventional chemical control options (Smith et al. 2016). In response to this critical threat there has been considerable investment in the development of alternative control strategies, involving the manufacture and release of genetically modified male mosquitoes (Harris et al. 2012, Carvalho et al. 2015, Aliota et al. 2016, Garcia et al. 2016, Joubert et al. 2016). Some of these mechanisms reduce the likelihood of *Ae. aegypti* transmitting viruses to humans (Aliota et al. 2016), while others aim to diminish wild populations (Harris et al. 2012, Carvalho et al. 2015). As these approaches rely on laboratory-reared transgenic males to adequately compete against their wild counterparts for mates, boosting their fitness will increase the effectiveness of vector control programs, and reduce the cost and the number of mosquitoes required for release (Alphey et al. 2010).

Until recently, the primary means of examining the fitness of *Ae. aegypti* in the laboratory was via comparison of measures such as mating competitiveness, insemination capacity, and survival between transgenic and wild-type males (Bargielowski et al. 2011, Massonnet-Bruneel et al. 2013, Patil et al. 2015). Some of these studies found that transgenesis has a negative effect on mating success (Bargielowski et al. 2011, Massonnet-Bruneel et al. 2013, Patil et al. 2015). Fitness costs associated with transgenesis may also be compounded by the negative effects of laboratory rearing. As laboratory-reared males are unlikely to be accustomed to the environmental pressures experienced in the wild, they may have a reduced capacity to exhibit normal mating behavior once released (Alphey et al. 2010, Bargielowski et al. 2011). Therefore, as well as improving genetic methods, there is an additional need to improve rearing procedures, in order to enhance the fitness of laboratory-reared males. This will result in males that are more effective at competing for mates in the wild.

One aspect of laboratory rearing that has been shown to influence the fitness of male mosquitoes is the quantity of food administered to mosquito larvae (Ponlawat and Harrington 2007, 2009, Ng’habi et al. 2008). Ng’habi et al. (2008) reported that male *Anopheles gambiae s.l. (Diptera: Culicidae*) reared on a ‘high’ quantity of fish food as larvae survive longer, while those reared on an ‘intermediate’ quantity, exhibit greater copulation success. Larval food availability has been shown to positively correlate with body size (Ng’habi et al. 2008; Ponlawat and Harrington 2007, 2009), and there is evidence to suggest that larger male *Anopheles* mosquitoes are more likely to participate in mating swarms in the field (Yuval et al. 1993, Sawadogo et al. 2013). There is surprisingly little corresponding data on the effect of rearing practices, including the quantity of larval food, on the fitness of *Ae. aegypti* mosquitoes. Most of the data currently available using *Aedes* as subjects, focus on post-copulatory aspects such as sperm capacity (Ponlawat and Harrington 2007, 2009; Helinski and Harrington 2011). We cannot assume that pre-copulatory aspects of mating success are similar between *Anopheles* and *Aedes* mosquitoes. These species exhibit divergent swarming and copulatory behaviors. While *Anopheles* mosquitoes swarm during twilight over visual markers (Yuval 2006, Diabate and Tripet 2015), *Aedes* mosquitoes swarm during the day in close proximity to a host (Hartberg 1971). Furthermore, *Anopheles* swarms are typically reported as larger than those of *Aedes* (Cator et al. 2011, Diabate and Tripet 2015).

To ensure that these mosquitoes will be successful in the wild they must survive long enough to mate, participate in mating swarms, and have the ability to copulate with females (Cabrera and Jaffe 2007, Alphey et al. 2010, Oliva et al. 2014). Here we investigated the effect of the quantity of larval food on adult survival, swarming activity and copulation success, in male *Ae. aegypti* mosquitoes. We found that larval food availability was positively correlated with body size. Larger males had greater longevity and greater swarming activity. However, we found an inconsistent effect of body size on male success in mating competition experiments. Our results have implications for optimizing the larval dietary conditions used in the breeding of male *Ae. aegypti* for vector control programs, by showing that multiple fitness measures, and experimental techniques should be considered.

## Materials and Methods

### Mosquito Rearing

All experiments were conducted on *Ae. aegypti* originating from Fort Myers, Florida (F8-12) (Bargielowski et al. 2013). Eggs for each experiment were hatched under a vacuum, provided with 10 mg of ground fish food (Cichlid gold, Hikari, Kasai, Japan), and held overnight at 26–28°C, 60–75.5% RH, and 14:10 (L:D) h cycle. This range of conditions remained the same throughout the rearing process and the subsequent experiments. As the quantity of larval food influences the development rate of *Ae. aegypti* (Couret et al. 2014), hatching dates were staggered, to ensure that the mosquitoes from all diet groups emerged around the same time. To ensure that the individuals from all diets were from the same mix of parental populations and clutches, eggs papers were split evenly between the diet groups. The day after hatching, first instar larvae were sorted into groups of approximately 200 larvae, and placed in trays containing 500 ml of distilled water. The diet treatments were then imposed. For the survival experiments, the trays were provided with either 0.1 or 1.0 mg of ground fish food per larva per day. For the swarming activity experiments, trays were provided with either 0.1 or 0.3 mg of ground fish food per larva per day. Lastly, for the mating competition experiments, trays were provided with either 0.1, 0.3, or 0.5 mg of ground fish food per larva per day. At pupation, individuals were placed separately in 10 ml falcon tubes plugged with cotton wool to allow for adult emergence. For all experiments wing-length was used as a proxy for body size. The wing was measured from the distal end of the alula to the tip of the wing, excluding the hairy fringe (Nasci 1990).

### Adult Survival

Females from each diet treatment were held in 19 cm^3^ plexiglass cages and were provided with 20% sucrose solution. Newly emerged adult males were aspirated into one of 16 cups covered with mesh lids, at a frequency of 10 males per cup. Four of these cups contained males from the 0.1 mg diet that would remain virgins, while a further four cups contained males from the 1.0 mg diet that would remain virgins. These males were to be used to establish the effect of the quantity of larval food on survival. Four cups contained males from the 0.1 mg diet that would be exposed to females, while the remaining four cups contained males from the 1.0 mg diet that would be exposed to females. These males were to be used to establish the effect of mating on survival, for the two diet groups. Both males and females were provided with a pad of cotton wool soaked in a 20% sucrose solution. After 5 d, the males to be exposed to females were aspirated individually into 15 ml falcon tubes. Half of these males were exposed to a female from the same diet as themselves, and half to a female from the other diet. They were held in the tubes for 24 h, and provided with 20% sucrose solution. At the end of this period the males were transferred back to their respective cups. Female reproductive tracts were dissected in 70% ethanol and visualized under a dissecting microscope. The presence of sperm in the spermathecae was established. This gave an overall measure of how many females mated per cup, but individual male mating status and survival was not tracked. Male mortality in cups was monitored daily for 30 d after being exposed to a female. The cups containing males which were not exposed to females, remained in their cups throughout the experiment, and mortality was also monitored for 30 d from the day that they were first put into the cups. Upon death the right wing of each male was measured to determine body size under a dissecting microscope. This experiment was carried out twice.

### Swarming Activity

Upon emergence males and females were placed in separate 19 cm^3^ plexiglass cages. Male cages were set up at a density of 20 males per cage, and were provided with 20% sucrose solution. Swarming observations began when males were between 36 and 48 h old. The night before swarming observations, the sucrose solution was removed to limit compensatory sugar feeding and ensure that any differences observed between treatment groups was due to larval diet alone. All swarming observations took place between 09:00 and 11:30 a.m. In most cases, cages containing males from the 0.1 mg and 0.3 mg diets were video recorded simultaneously. Paper towel was taped around the sides of the cages to ensure the mosquitoes could not see each other, and to minimize the effects of any disturbances in the room. To stimulate swarming, a worn sock (12 h prior, K.M.) was introduced into the two cages. Firstly, males were recorded swarming alone. Afterwards, the males in these cages were left to rest for 36 h, with access to 20% sucrose solution, and then an additional 12 h without sucrose solution. Female cues were then provided to males by placing a cage containing 20 females (10 from each larval diet) on top of each male cage, so that the two large mesh windows faced each other. In several trials the order was reversed such that males were recorded firstly with females present, allowed to rest for 48 h, and then recorded alone.

A male was considered to be swarming when it exhibited the stereotyped figure of eight flying pattern (Clements 1992). Male swarming behaviors were video recorded for 12 min. Videos were analyzed, and the number of males swarming every 30 s and the maximal number of males participating in the swarm at the peak were determined. The date of observation and male age was noted for all swarming cages throughout the study. Two to three mosquitoes from each cage of males were killed after the swarming experiments, the right wings were dissected, and their lengths were measured under a dissecting microscope. There were a total of 35 cages containing males from the 0.1 mg diet, and 26 cages containing males from the 0.3 mg diet that were recorded swarming alone, and a total of 25 cages containing males from the 0.1 mg diet and 16 cages containing males from the 0.3 mg diet recorded swarming next to females 48 h later. There were a total of five cages containing males from the 0.1 mg diet, and five cages containing males from the 0.3 mg diet recorded next to females first, and then all of these cages were recorded alone 48 h later.

### Mating Competition

Upon emergence males and females were placed in separate 19 cm^3^ plexiglass cages containing 20% sucrose solution. The sucrose was removed 24 h prior to the mating competition experiments. The experiments took place 48–72 h after emergence. Mosquitoes were marked so that their diet treatment could be identified after the competition experiments. Two different marking methods were used. First, males and females from the different larval diets were placed in sex specific cups with mesh lids. A syringe was filled up to the 0.4 ml marking with fluorescent dust, and was used to pump the dust into the cup to create a cloud (bulb duster method, Dickens and Brant 2014). Four males from each of the three diet treatments were then aspirated into each 19 cm^3^ plexiglass cage for the mating competition experiments. For the second method, mosquitoes were anaesthetized on ice for 3–5 min. They were then moved to petri dishes and a 2 cm water color brush, which had been immersed in one of the three dust colors, was used to sprinkle dust on to males and females (paint method, Dickens and Brant 2014). Again, four males from each of the three larval diets were aspirated into each 19 cm^3^ plexiglass cage for the mating competition experiments. Mating experiments began after the mosquitoes had recovered from the anaesthesia. For the bulb duster method, blue and pink fluorescent dust from Swanda Inc., Stalybridge, United Kingdom was used, and yellow fluorescent dust from Killgerm, Ossett, United Kingdom. For the paint method, the same blue and pink dusts were used, but a yellow fluorescent dust also from Swanda Inc. was used instead. In all cases the three dusts were alternated evenly between the diet groups throughout the experiments, to control for an effect of dust type on copulation success.

Once males were swarming, one female from each of the three diet groups were aspirated into the cage. When copula formation was observed the pair was aspirated out of the cage while in copula, and put into a labeled cup containing a mesh lid. A copula was counted when the male was observed to clasp his genitalia to the female genitalia. The trial was halted after all three females in each cage mated. The mated pairs were later observed under a dissecting microscope to view the fluorescent dust and hence, identify the larval diet. After death the right wing was removed and the wing-length was measured under a dissecting microscope. The experiment using the bulb duster method was repeated three times, with 21, 18, and 15 trials, respectively per replicate (54 trials all together). The experiment when using the paint method was carried out twice, with 30 trials in each replicate (60 trials all together).

### Sperm Transfer

To establish the percentage of observed copulations in which sperm is transferred, mosquitoes were reared using the same methodology outlined in the ‘Mosquito Rearing’ section above, and were provided with 0.3 mg of ground fish food per larva per day. At emergence males and females were placed in separate 19 cm^3^ plexiglass cages containing 20% sucrose solution, which was removed 24 h prior to mating. The experiments took place 48–72 h after emergence. A male and female were aspirated into a 19 cm^3^ plexiglass cage. When copula formation was observed the pair was aspirated out of the cage while in copula and immediately separated. The female was put into a cup containing a mesh lid and the male was discarded. A fresh male and female were placed into the cage for the next trial. This procedure was repeated until 21 copulas had been collected. Mated females were anaesthetized on ice. The reproductive tracts were dissected out of the females in 1% phosphate buffered saline under a dissecting microscope, to extract the spermathecae. Spermathecae were ruptured by placing a glass coverslip on top of them and the presence of sperm was checked under a compound scope.

### Statistical Analysis

All data was deposited into Dryad (doi:10.5061/dryad.n26r).

#### Adult Survival

Two-tailed Student’s *t*-tests were used to establish whether there was a significant difference between the wing-lengths of males from the 0.1 mg and the 1.0 mg larval diets. The effect of diet treatment, mating treatment, replicate and their interaction on male survival was tested using a Cox Regression (Field 2009). Data from 320 males were used for the analysis (80 from each diet/mating treatment). The model was established using SPSS IBM version 23 (Field 2009).

#### Swarming Activity

Two-tailed Student’s *t*-tests were used to establish whether there was a significant difference between the wing-lengths of males from the 0.1 mg and the 0.3 mg larval diets. To assess the effect of diet treatment, female presence/absence, age of males, the date and time of recording, and the interaction between these variables, on the number of males swarming every 30 s over 12 min, a Generalized Linear Mixed Effect Model (GSLMM) fit with a log-linear distribution was used (Field 2009). The effect of diet treatment on the maximum number of swarming males and the time until maximum swarming per cage, was established using a General Linear Model (GLM) (Field 2009). Data from 122 swarming trials were used in the analysis for both models (0.1 mg females absent = 40 trials; 0.3 mg females absent = 31 trials; 0.1 mg females present = 30 trials; 0.3 mg females present = 21 trials). The models were created using R software version 3.1.2 (R Core Team 2014).

#### Mating Competition

One-way ANOVAs were used to establish whether there was a significant difference between the wing-lengths of males from the 0.1 mg, 0.3 mg, and 0.5 mg larval diets. The effect of diet treatment, dust type, and replicate along with their interactions on male copulation success was tested using binomial Generalized Linear Models (GSLMs) (Field 2009). A total of 54 and 60 mating competition trials were included in the analysis for the bulb duster method and the paint method, respectively. A total of three replicates were included for the bulb duster method, and two for the paint method. Chi-squared tests were used to establish whether the larval diet of the females influences the likelihood of being the first of the three females to mate. The presence of size assortative mating was tested using poisson GSLMs (Field 2009). The models were established using R Studio software version 0.99.902 (R Core Team 2014).

## Results

### Adult Survival

**Fig. 1.**
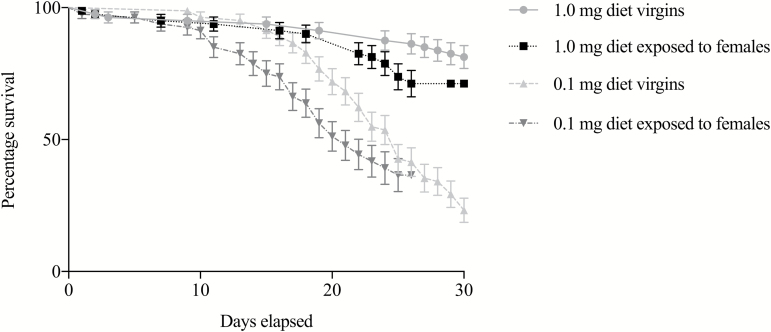
Effect of the quantity of larval food and mating on male survival. Each line represents 80 male mosquitoes (two replicates each, including 40 males per treatment). The error bars represent ± 1 SE.

Males from the 1.0 mg diet had a significantly larger body size than those from the 0.1 mg diet (Student’s *t*-test; *t* = 22.83; df = 148.15; *P* < 0.001; Table 1). Mating alone did not have a significant effect on survival (Cox Regression; Wald; χ^2^ = 0.01; df = 1; *P* = 0.99; Fig. 1). Our dissections indicated that mating did indeed occur for the partnered males and females, with greater than 50% of females mating in all cups. There was a significant effect of larval diet on survival, with males from the 1.0 mg diet exhibiting higher survival over 30 d compared to the males from the 0.1 mg diet (Cox Regression; Wald; χ^2^ = 46.79, df = 1; *P* < 0.001; Fig. 1). There was no significant interaction between diet treatment and mating treatment (Cox Regression; Wald; χ^2^ = 0.61; df = 1; *P* = 0.10; Fig. 1).

**Table 1.** The effect of the quantity of larval food on adult wing-length for males involved in the adult survival, swarming activity and mating competition experiments

Experiment	Larval diet (mg larva/d)	Wing-length (mm)
Adult survival	0.1	1.78 ± 0.014
	1.0	2.17 ± 0.011
Swarming activity	0.1	2.21 ± 0.020
	0.3	2.45 ± 0.017
Mating competition (bulb duster method)	0.1	1.85 ± 0.006
	0.3	2.03 ± 0.006
	0.5	2.14 ± 0.008
Mating competition (paint method)	0.1	1.86 ± 0.008
	0.3	2.06 ± 0.006
	0.5	2.18 ± 0.006

### Swarming Activity

**Fig. 2.**
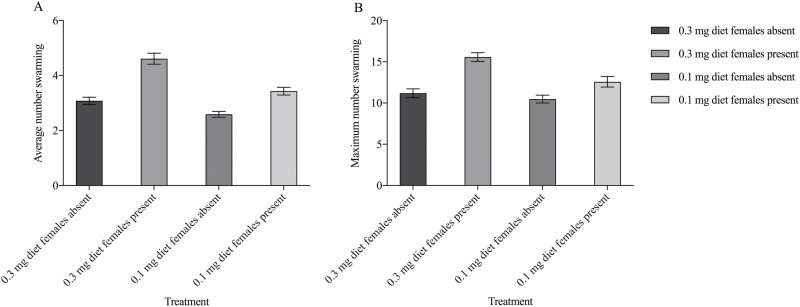
(A) Effect of the quantity of larval food and female presence on the average number of males swarming. (B) Effect of the quantity of larval food and female presence on the maximum number of males swarming. Error bars represent ± 1 SE. There were a total of 31 trials for ‘0.3 mg diet females absent’, 21 trials for ‘0.3 mg diet females present’, 40 trials for ‘0.1 mg diet females absent’ and 30 trials for ‘0.1 mg diet females present’.

Males from the 0.3 mg larval diet had a significantly larger body size than those from the 0.1 mg diet (Student’s *t*-test; *t* = 9.12; df = 172.3; *P* < 0.001; Table 1). Significantly more males swarmed on average in cages containing males from the 0.3 mg diet (3.08 ± 0.13 males) than the 0.1 mg diet (2.59 ± 0.11 males) (diet; GSLMM; *F* = 7.21; df1 = 1; df2 = 3045; *P* = 0.007; Fig. 2). In addition, males from the 0.3 mg diet exhibited an increase in the average number of males swarming when presented with females that was unmatched by males from the 0.1 mg diet (diet × female presence; GSLMM; *F* = 30.38; df1 = 2; df2 = 3045; *P* < 0.001; Fig. 2). When females were presented there was an average of 4.61 ± 0.20 males swarming from the 0.3 mg diet, and a lesser average of 3.43 ± 0.14 males swarming from the 0.1 mg diet. Separate from this effect, older males had a greater increase in swarming when females were presented (age × female presence; GSLMM; *F* = 110.93; df1 = 1; df2 = 3045; *P* < 0.001).

Cages containing males from the 0.3 mg diet also had a significantly higher maximum number of males swarming at their peak (11.19 ± 0.54 males), than males from the 0.1 mg diet (10.48 ± 0.48 males) (diet; GLM; χ^2^ = 6.13; df = 1; *P* = 0.013; Fig. 2). The presence of females also significantly increased the maximum number of males swarming (female presence; GLM; χ^2^ = 22.43, df = 1; *P* < 0.001; Fig. 2). Again, cages containing males from the 0.3 mg diet exhibited significantly higher maximums when females were present in comparison to the 0.1 mg diet (diet × female presence; GLM; χ^2^ = 4.17; df = 3; *P =* 0.041; Fig. 2). When females were presented there was a maximum of 15.57 ± 0.53 males swarming from the 0.3 mg diet, and a lesser maximum of 12.58 ± 0.64 males swarming from the 0.1 mg diet.

### Mating Competition

**Fig. 3.**
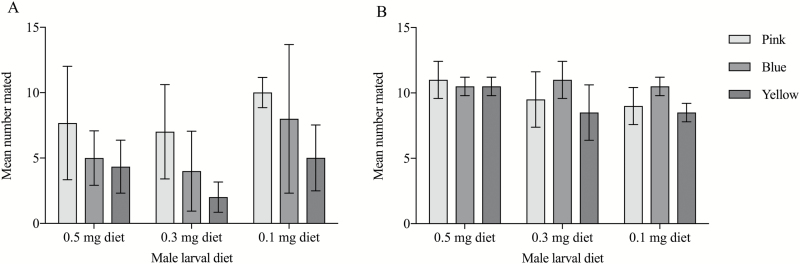
(A) Effect of the quantity of larval food and dust type on the mean number of males mated using the bulb duster method. Error bars represent ± 1 SE (54 mating competition trials). (B) Effect of the quantity of larval food and dust type on the mean number of males mated using the paint method. Error bars represent ± 1 SE (60 mating competition trials).

There was a significant difference between the body sizes of males from the three diet treatments. Males fed on a greater quantity of larval food were larger (ANOVA; bulb duster method; *F* = 465; df1= 2; df2 = 600; *P* < 0.001; ANOVA; paint method; *F* = 583; df1 = 2; df2 = 686; *P* < 0.001; Table 1). When males and females were marked using the bulb duster method an effect of male diet treatment on copulation success was consistently found (diet; GSLM; χ^2^ = 12.17; df = 2; *P =* 0.002; Fig. 3). Out of all of the mating’s 32.08% involved a male from of the 0.5 mg diet, 24.53% involved a male from the 0.3 mg diet, and 43.4% involved a male from the 0.1 mg diet. The effect of male diet treatment was significant, despite a significant effect of dust type (dust type; GSLM; χ^2^ = 20.37; df = 2; *P* < 0.001; Fig. 3). The yellow dust, which was a different brand to the other dusts, appeared to negatively affect the mating behavior of the mosquitoes. When the males and females were marked using the paint method there was no effect of dust type on copulation success (dust type; GSLM; χ^2^ = 0.78; df = 2; *P* = 0.86; Fig. 3), in which a different yellow dust was used. There was also no effect of larval diet when using the paint method (diet; GSLM; χ^2^ = 0.52; df = 2; *P* = 0.94; Fig. 3). The percentage of mating’s involving males from the 0.1 mg and 0.3 mg diets were both 33.15%, while 33.71% of mating’s involved males from the 0.5 mg diet. There was no effect of female larval diet on the likelihood of being the first of the three females to mate (Chi-squared test; bulb duster method; χ^2^ = 2.17; df = 2; *P* = 0.34; Chi-squared test; paint method; χ^2^ = 1.91; df = 2; *P* = 0.38). In both experiments, there was no evidence of size assortative mating (GSLM; bulb duster method; χ^2^ = 0.43; df = 1; *P* = 0.51; GSLM; paint method; χ^2^ = 0.80; df = 2; *P* = 0.67).

### Sperm Transfer

Observations of copulas were highly indicative of sperm transfer. Out of 21 mated females, 20 (95.24 ± 4.76%) contained sperm in the spermathecae.

## Discussion

An evidence-based approach to improving male fitness would be beneficial for efforts to control wild *Ae. aegypti* populations using transgenic males mass-reared in the laboratory. While previous work has addressed the effect of larval diet on adult male sperm production and transfer in laboratory reared *Ae. aegypti* (Ponlawat and Harrington 2007, 2009; Helinski and Harrington 2011), there is very little published data on the effect of larval diet on pre-copulatory aspects of male mating success in this species. Considering that females are believed to be predominantly monandrous (Clements 1999, but see Helinski et al. 2012), these aspects of mating behavior may be of great importance for facilitating release success. In this study we explored the effect of larval food availability, which was found to be positively correlated with adult body size, on the survival, swarming activity and copulation success of adult male *Ae. aegypti* mosquitoes. We found that increasing the body size enhanced the survival and swarming activity of males. However, it did not increase the likelihood of them successfully copulating within a swarm.

Similar to what we observed here in *Ae. aegypti*, a positive effect of larval diet and adult body size on male survival (Ng’habi et al. 2008) and swarming activity ( Yuval et al. 1993, Yuval et al. 1998, Sawadogo et al. 2013) has been reported in numerous other mosquito species and dipterans. These observations may be explained by the greater energy reserves of individuals provided with a more nutritious diet (Ng’habi et al. 2008). Interestingly, there was no significant difference in survival between virgin males and those males exposed to females. This is surprising as mating is generally considered a costly process (South et al. 2009, Bargielowski et al. 2011). It is possible that in *Ae. aegypti* mating is not as costly as previously thought. However, the fact that not all males exposed to females mated, may explain that while there is a trend towards a lower survival of males exposed to females in comparison to virgins, the difference is on average not significant. Although we are unable to test the effect of individual male mating status on survival, since females were pooled together after the experiments to check for spermathecae.

Swarming activity was maximized when there was a female stimulus even though males could not access females. This indicates that cues that may be perceived by the males without physical contact, can alter swarming behavior. Larger males increased their swarming activity to a greater extent than smaller males, which may again be a result of the higher energy reserves of larger males (Ng’habi et al. 2008). However, an alternative hypothesis is that the outcome of the swarming experiments may be a result of female preference. If females prefer larger males, they may be more active and solicit swarming behavior from these males to a greater extent (Sawadogo et al. 2013).

A number of studies have demonstrated that larger male and female mosquitoes are more fecund, which may explain an innate preference for a larger mate (Okanda et al. 2002; Ponlawat and Harrington 2007, 2009; Helinski and Harrington 2011). However, we did not find a consistent effect of larval diet and male body size on the copulation success between competition experiments. When utilizing the bulb duster method, we found that smaller males had the greatest copulation success. This type of effect has been reported in other swarming insects such as midges and mayflies, in which a smaller size allows for greater flight agility, which would enable a male to reach a female more easily in a frenzied swarm (Crompton et al. 2003, Neems et al. 1998, Peckarsky et al. 2002). In this same experiment we found a significant effect of the dust type on copulation success. The yellow dust, which was a different brand to the others, had a negative effect on mating behavior. However, the different types of dust were alternated evenly between the three diet groups throughout the experiments to control for this effect, and when dust type was controlled for, the significant effect of the diet still existed. To confirm our results, we repeated the same experiments using a different marking method. When utilizing the paint method, in which we used a different brand of yellow dust, there was no effect of the dust type on copulation success, but there was also no effect of body size. A lack of a relationship between male body size and copulation success in *Ae. aegypti*, was previously observed in mating experiments by Cator and Zanti (2016), in which one large or small tethered female was given the opportunity to mate with one of five free flying large or small males.

The contrasting results between the two methodologies may be a result of the marking techniques used. It is possible that different marking techniques, may affect the sensory organs to different extents, and may in some instances affect their ability to differentiate between potential mates based on size (Dickens and Brant 2014). The extent of this hindrance may be due to the quantity of dust that adheres to the sensory organs.

It is also important to note that while our data indicated that observed copulation in these mating competition experiments are generally indicative of sperm transfer, there may be a differential ‘false’ copula rate dependent on larval diet. Small males for example, may not be as successful at transferring sperm to females as large males during copulation. Our focus here was on pre-copulatory mating success. It has been established by others that male size can affect the amount of sperm transferred to females (Ponlawat and Harrington 2007, 2009), but future experiments could explore whether male larval diet alters transfer success rates.

Our results indicate that although larger males may be more likely to participate in a swarm, as a result of their greater swarming activity and survival, they may not necessarily have an increased copulation success once in a swarm. While this group of experiments does allow us to generally investigate the effect of larval diet on adult mating success, the fact that the diet treatments are not identical between experiments limits our ability to comment on trade-offs between different traits associated with diet effects. Additionally, given the inconsistent effect of marking on mating competition experiments we must be cautious in interpreting our results. In the future, it would be useful to explicitly test if, and in what way different marking methods bias the outcomes of fitness experiments.

Furthermore, it would be beneficial to establish the effect of other factors such as the type of larval food, rearing density, temperature and adult diet, and how they interact with the effect of the quantity of larval food, to influence male fitness (Tun-Lin et al. 2000, Bellini et al. 2014, Couret et al. 2014). For example, we deprived adults of sugar prior to experiments. This was done to isolate the effect of larval diet from any separate or interactive effect with adult diet. The evidence for sugar feeding in adult *Ae. aegypti* in nature is varied (Spencer et al. 2005, Bellini et al. 2014). However, future work could investigate how sugar feeding, and its interaction with larval diet influence male swarming activity and mating competitiveness.

Moving forward, it is also important that fitness experiments are conducted in the field, to observe whether the same conclusions are reached as those observed in the laboratory. Wild or laboratory-released resting, swarming and mating *Ae. aegypti* males should be collected and their body size established, to determine how body size influences swarming activity and the likelihood of mating in the field (Yuval et al. 1993, Sawadogo et al. 2013). This is an important next step, because male mosquitoes to be used for vector control programs, will eventually be released into the field. These males must have the ability to compete effectively for females, with added environmental pressures that they are not accustomed to.

Our study reveals the importance of testing fitness using a holistic approach, taking into account a range of different life history traits. The study further demonstrates the importance of testing different experimental techniques, when establishing how rearing practices can be altered to produce adult males with the best chance at out-competing wild populations of *Ae. aegypti.* Laboratory studies such as these, as well as further studies in the field, will improve the efficiency of vector control programs, and reduce the transmission of many lethal human diseases.

## Data Availability Statement

Data from this study are available from the Dryad Digital Repository: http://dx.doi.org/10.5061/dryad.n26r0

## References

[CIT0001] Aliota M. T., Peinado S. A., Velez I. D., and Osorio J. E. 2016 The *w*Mel strain of *Wolbachia* reduces transmission of Zika virus by *Aedes aegypti*. Sci. Rep. 6: 28792. 27364935 10.1038/srep28792 PMC4929456

[CIT0002] Alphey L., Benedict M., Bellini R., Clark G. G., Dame D. A., Service M. W., and Dobson S. L. 2010 Sterile-insect methods for control of mosquito-borne diseases: an analysis. Vector. Borne. Zoonotic. Dis. 10: 295–311. 19725763 10.1089/vbz.2009.0014 PMC2946175

[CIT0003] Bargielowski I., Alphey L., and Koella J. C. 2011 Cost of mating and insemination capacity of a genetically modified mosquito *Aedes aegypti* OX513A compared to its wild type counterpart. PLoS One. 6: e26086. 22022518 10.1371/journal.pone.0026086 PMC3191171

[CIT0004] Bargielowski I. E., Lounibos L. P., and Carrasquilla M. C. 2013 Evolution of resistance to satyrization through reproductive character displacement in populations of invasive dengue vectors. PNAS. 110: 2888–2892. 23359710 10.1073/pnas.1219599110 PMC3581888

[CIT0005] Bellini R., Puggioli A., Balestrino F., Brunelli P., Medici A., Urbanelli S., and Carrieri M. 2014 Sugar administration to newly emerged *Aedes albopictus* males increases their survival probability and mating performance. Acta. Trop. 132S: S116–S123. 10.1016/j.actatropica.2013.11.022 24299923

[CIT0006] Cabrera M., and Jaffe K. 2007 An aggregation pheromone modulates lekking behaviour in the vector mosquito *Aedes aegypti* (Diptera: Culicidae). J. Am. Mosq. Control. Assoc. 23: 1–10. 17536361 10.2987/8756-971X(2007)23[1:AAPMLB]2.0.CO;2

[CIT0007] Carvalho D. O., McKemey A. R., Garziera L., Lacroix R., Donnelly C. A., Alphey L., Malavasi A., and Capurro M. L. 2015 Suppression of a field population of *Aedes aegypti* in Brazil by sustained release of transgenic male mosquitoes. PLoS. Negl. Trop. Dis. 9: e0003864. 26135160 10.1371/journal.pntd.0003864 PMC4489809

[CIT0008] Cator L. J., Arthur B. J., Ponlawat A., and Harrington L. C. 2011 Behavioral observations and sound recordings of free-flight mating swarms of *Ae. aegypti* (Diptera: Culicidae) in Thailand. J. Med. Entomol. 48: 941–946. 21845959 10.1603/me11019 PMC4948640

[CIT0009] Cator L. J., and Zanti Z. 2016 Size, sounds, and sex: Interactions between body size and harmonic convergence signals determine mating success in *Aedes aegypti*.Parasit. Vectors. 9: 622–633. 27906074 10.1186/s13071-016-1914-6 PMC5133739

[CIT0010] Clements A. N 1992 The biology of mosquitoes: volume 2 sensory reception and behaviour. CABI Publishing, Wallingford, United Kingdom.

[CIT0011] Clements A. N 1999 The biology of mosquitoes. CABI Publishing, Wallingford, United Kingdom.

[CIT0012] Couret J., Dotson E., and Benedict M. Q. 2014 Temperature, larval diet, and density effects on development rate and survival of *Aedes aegypti* (Diptera: Culicidae). PLoS One. 9: e87468. 24498328 10.1371/journal.pone.0087468 PMC3911954

[CIT0013] Crompton B., Thomason J. C., and McLachlan A. 2003 Mating in a viscous universe: the race is to the agile, not the swift. Proc. R. Soc. Lond. B. Biol. Sci. 270: 1991–1995. 10.1098/rspb.2003.2477 PMC1691468 14561286

[CIT0014] Diabate A. and Tripet F. 2015 Targeting male mosquito mating behaviour for malaria control. Parasit. Vectors. 8: 347. 26113015 10.1186/s13071-015-0961-8 PMC4485859

[CIT0015] Dickens B. L., and Brant H. L. 2014 Effects of marking methods and fluorescent dusts on *Aedes aegypti* survival. Parasit. Vectors. 7: 65. 24520937 10.1186/1756-3305-7-65 PMC3937048

[CIT0016] Field A 2009 Discovering statistics using IBM SPSS. Sage Publications, London, United Kingdom.

[CIT0017] Garcia G. D. A.L.M. dos Santos B., D. A.M. Villela, and Maciel-de-Freitas R. 2016 Using *Wolbachia r*eleases to estimate *Aedes aegypti* (Diptera: Culicidae) population size and survival. PLoS ONE. 11: e0160196. 27479050 10.1371/journal.pone.0160196 PMC4968812

[CIT0018] Harris A. F., McKemey A. R., Nimmo D., Curtis Z., Black I., Morgan S. A., Oviedo M. N., Lacroix R., Naish N., Morrison N. I., 2012 Successful suppression of field mosquito population by sustained release of engineered male mosquitoes. Nature. Biotechnol. 30: 828–830. 22965050 10.1038/nbt.2350

[CIT0019] Hartberg W. K 1971 Observations on the mating behaviour of *Aedes aegypti* in nature. Bull. World. Health. Organ. 45: 847–850. 5317018 PMC2428001

[CIT0020] Helinski M. E. H., and Harrington L. C. 2011 Male mating history and body size influence female fecundity and longevity of the dengue vector *Aedes aegypti*.J. Med. Entomol. 48: 202–211. 21485355 10.1603/me10071 PMC4182911

[CIT0021] Helinski M. E. H., Valerio L., Facchinelli L., Scott T. W., Ramsey J., and Harrington L. C. 2012 Evidence of polyandry for *Aedes aegypti* in semifield enclosures. Am. J. Trop. Med. Hyg. 86: 635–641. 22492148 10.4269/ajtmh.2012.11-0225 PMC3403777

[CIT0022] Joubert D. A., Walker T., Carrington L. B., De Bruyne J. T., Kien D. H. T., Hoang N. L. T., N. V.V. Chau I. Iturbe-Ormaetxe C. P. Simmons, and O’Neill S. L. 2016 Establishment of *Wolbachia* Superinfection in *Aedes aegypti* mosquitoes as a potential approach for future resistance management. PLoS. Pathog. 12: e1005434. 26891349 10.1371/journal.ppat.1005434 PMC4758728

[CIT0023] Massonnet-Bruneel B., Corre-Catelin N., Lacroix R., Lees R. S., Hoang K. P., Nimmo D., Alphey L., and Reiter P. 2013 Fitness of transgenic mosquito *Aedes aegypti* males carrying a dominant lethal genetic system. PLoS ONE. 8: e62711. 23690948 10.1371/journal.pone.0062711 PMC3653897

[CIT0024] Nasci R. S 1990 Relationship of wing length to adult dry weight in several mosquito species (Diptera: Culicidae). J. Med. Entomol. 27: 716–719. 2388250 10.1093/jmedent/27.4.716

[CIT0025] Neems R. M., Lazarus J., and McLachlan A. J. 1998 Lifetime reproductive success in a swarming midge: trade-offs and stabilizing selection for male body size. Behav. Ecol. 9: 279–286.

[CIT0026] Ng’habi K. R., Huho B. J., Nkwengulila G., Killeen G. F., B. G.J. Knols, and Ferguson H. M. 2008 Sexual selection in mosquito swarms: may the best man lose?Anim. Behav. 76: 105–112.

[CIT0027] Okanda F. M., Dao A., Njiru B. N., Arija J., Akelo H. A., Toure Y., Odulaja A., Beier J. C., Githure J. I., Yan G., 2002 Behavioural determinants of gene flow in malaria vector populations: *Anopheles gambiae* males select large females as mates. Malar. J. 1: 10. 12296972 10.1186/1475-2875-1-10 PMC140138

[CIT0028] Oliva C. F., Damiens D., and Benedict M. Q. 2014 Male reproductive biology of *Aedes* mosquitoes. Acta. Trop. 132: S12–S19. 24308996 10.1016/j.actatropica.2013.11.021

[CIT0029] Patil P. B., Reddy B. P. N., Gorman K., K. V.S. Reddy S. R. Barwale U. B. Zehr D. Nimmo N. Naish, and Alphey L. 2015 Mating competitiveness and life-table comparisons between transgenic and Indian wild-type *Aedes aegypti* L. Pest Manag Sci. 71: 957–965. 25078081 10.1002/ps.3873 PMC4657483

[CIT0030] Peckarsky B. L., McIntosh A. R., Caudill C. C., and Dahl J. 2002 Swarming and mating behaviour of a mayfly *Baetis bicaudatus* suggest stabilizing selection for male body size. Behav. Ecol. Sociobiol. 51: 530–537.

[CIT0031] Petersen L. R., Jamieson D. J., Powers A. M., and Honein M. A. 2016 Zika Virus. N. Engl. J. Med. 374: 1552–1563. 27028561 10.1056/NEJMra1602113

[CIT0032] Ponlawat A., and Harrington L. C. 2007 Age and body size influence male sperm capacity of the dengue vector *Aedes aegypti* (Diptera: Culicidae). J. Med. Entomol. 44: 422–426. 17547226 10.1603/0022-2585(2007)44[422:aabsim]2.0.co;2

[CIT0033] Ponlawat A., and Harrington L. C. 2009 Factors associated with male mating success of the dengue vector mosquito, *Aedes aegypti*.Am. J. Trop. Med. Hyg. 80: 395–400. 19270288

[CIT0034] R Core Team. 2014 R: A language and environment for statistical computing. R Foundation for Statistical Computing, Vienna, Austria.

[CIT0035] Sawadogo S. P., Diabate A., Toe H. K., Sanon A., Lefevre T., Baldet T., Gilles J., Simard F., Gibson G., Sinkins S., and Dabire R. 2013 Effects of age and size on *Anopheles gambiae s.s*. male mosquito mating success. J. Med. Entomol. 50: 285–293. 23540115 10.1603/me12041

[CIT0036] Smith L. B., Kasai S., and Scott J. G. 2016 Pyrethroid resistance in *Aedes aegypti* and *Aedes albopictus*: important mosquito vectors of human diseases. Pest. Biochem. Physiol. 133: 1–12. 10.1016/j.pestbp.2016.03.005 27742355

[CIT0037] South S. H., Steiner D., and Arnqvist G. 2009 Male mating costs in a polygynous mosquito with ornaments expressed in both sexes. Proc. R. Soc. Lond. B. Biol. Sci. 276: 3671–3678. 10.1098/rspb.2009.0991 PMC2817312 19640881

[CIT0038] Spencer C. Y., Pendergast T. H. IV, and Harrington L. C 2005 Fructose variation in the dengue vector, *Aedes aegypti*, during high and low transmission seasons in the Mae Sot region of Thailand. J. Am. Mosq. Control. Assoc. 21: 177–181. 16033119 10.2987/8756-971X(2005)21[177:FVITDV]2.0.CO;2

[CIT0039] Tolle M. A 2009 Mosquito-borne diseases. Curr. Probl. Pediatr. Adolesc. Health. Care. 39: 97–140. 19327647 10.1016/j.cppeds.2009.01.001

[CIT0040] Tun-Lin W., Burkot T. R., and Kay B. H. 2000 Effects of temperature and larval diet on development rates and survival of the dengue vector *Aedes aegypti* in north Queensland, Australia. Med. Vet. Entomol. 14: 31–37. 10759309 10.1046/j.1365-2915.2000.00207.x

[CIT0041] Yuval B 2006 Mating systems of blood-feeding flies. Annu. Rev. Entomol. 51: 413–440. 16332218 10.1146/annurev.ento.51.110104.151058

[CIT0042] Yuval B., Wekesa J. W., and Washino R. K., 1993 Effect of body size on swarming behavior and mating success of male *Anopholes freeborni* (Diptera: Culicidae). J. Insect. Behav. 6: 333–342.

[CIT0043] Yuval B., Kaspi R., Shloush S., and Warburg M. S. 1998 Nutritional reserves regulate male participation in Mediterranean fruit fly leks. Ecol. Entomol. 23: 211–215.

